# Temporarily Reversing Warfarin With Low-Dose 4-Factor Prothrombin Complex Concentrate in Left Ventricular Assist Device Patients Undergoing an Invasive Procedure

**DOI:** 10.1177/10600280241248172

**Published:** 2024-04-27

**Authors:** Byron Stevenson, A. Joshua Roberts, William E. Dager

**Affiliations:** 1 Davis Medical Center, University of California, Sacramento, CA, USA

**Keywords:** prothrombin complex concentration, left ventricular assist device, coagulation, anticoagulation

## Abstract

**Background:**

American Association for Thoracic Surgery and The International Society for Heart and Lung Transplantation (AATS/ISHLT) guidelines recommend warfarin in patients with continuous-flow left ventricular assist devices (LVADs) to reduce the risk of device thrombosis and systemic embolization. Left ventricular assist device patients often undergo elective and emergent procedures that require interrupted anticoagulation. Data and experience vary on the optimal strategy to rapidly reverse warfarin in LVAD patients when an emergent procedure is planned.

**Objective:**

The purpose of this study was to describe the use of 4-factor prothrombin complex concentrate (PCC4) for warfarin reversal in patients with LVADs undergoing elective and emergent procedures.

**Methods:**

This retrospective, single-center, cohort review describes the use of PCC4 in patients with LVADs who require warfarin reversal for elective or emergent procedures. The primary outcome was a composite incidence of pump thrombosis, venous thromboembolism, and ischemic stroke within 30 days of PCC4 administration.

**Results:**

In total, 14 patients received 17 administrations of PCC4. One patient received 3 administrations, and 1 other patient received 2 administrations during separate encounters. The median dose was 500 units or 6.6 units/kg (range = 4.2-14.1 units/kg). Of the PCC4 administrations, 82% (14/17) were for low bleed risk procedures and 76% (13/17) were for elective procedures. There were no cases of pump thrombosis, venous thromboembolism, or stroke within 30 days of the procedure.

**Conclusions and Relevance:**

Low-dose PCC4 appears to be a safe and effective temporary reversal strategy for patients with LVADs undergoing low-bleed risk elective procedures.

## Introduction

Patients with durable, continuous-flow, left ventricular assist devices (LVADs) are at risk of device thrombosis and systemic embolization.^1^ Reasons for this are multifactorial in nature. Sheer stress and heat generated from device rotation, both in axial and centrifugal rotor design, are the most common device related drivers for pump thrombus formation.^2^ Inadequate pump pocket depth and inflow cannula angulation during surgical implantation have also been associated with pump thrombosis.^3^ In addition, mismanaged antithrombotic therapy is commonly observed in this population. This can be particularly detrimental to heart failure patients given elevations in plasma levels of von Willebrand factor, P-selectin, vascular endothelial growth factor (VEGF), tumor necrosis factor alpha (TNF-α), and interleukin-1 (IL-1).^4^ American Association for Thoracic Surgery/International Society for Heart Lung Transplantation (AATS/ISHLT) guidelines recommend oral anticoagulation with warfarin to an international normalized ratio (INR) goal of 2–3 for patients with continuous-flow LVADs.^5,6^ Typically, it is desirable to limit periods of INR values below target to minimize the risk of pump thrombosis.

Left ventricular assist device patients often undergo elective and urgent/emergent procedures that require temporary interruption of anticoagulation. Normalization of coagulation is often preferred to avoid procedural complications, including bleeding and the need for transfusions. Early procedural bleeding is associated with a nearly 20% increased risk for mortality.^7^ Strategies to reverse warfarin’s effects include withholding therapy and bridging with heparinoids, reversing warfarin directly with vitamin K, or utilizing concentrated blood clotting factor products. Holding warfarin and awaiting INR normalization may not be practical or desirable when a patient requires an urgent or emergent procedure, or reduced level of anticoagulation. Administration of vitamin K can present challenges when attempting to achieve rapid, temporary reversal and subsequently lead to delays when reinitiating warfarin time to target INR. Such situations may lead to anticoagulation bridging with either unfractionated (UF) heparin or a low-molecular weight heparin (LMWH). A low-dose vitamin K strategy to consider in LVAD patients was previously explored by us to limit the duration and intensity of vitamin K reversal.^8^

Four-factor prothrombin complex concentrate (PCC4) is effective at achieving rapid, temporary reversal of warfarin.^9,10^ Data and experience on using PCC4 to temporarily reverse the INR without precipitating thrombotic complications in patients with LVADs undergoing procedures are lacking. Currently, PCC4 has Food and Drug Administration (FDA)-approved indication for warfarin reversal in patients with acute major bleeding or who need for an urgent surgery/invasive procedure.^11,12^ Manufacturer recommended weight-based dosing depends on the pretreatment INR, with one of 3 recommended doses ranging from 25 units/kg to 50 units/kg (maximum 5000 units) with the intent for full reversal. However, FDA-approved dosing and related studies did not take into account patients with LVADs. Given the procoagulant effects of PCC4, the desire for limited or partial short-term reversal of warfarin’s anticoagulant effects, and the thrombotic concerns with LVADs, recommended doses for non-LVAD patients could potentiate thrombotic events when applied to the LVAD population.^13^ Based on initial requests from physicians, including our heart failure specialist, to provide a temporary reversal and limit the duration of hospital stay to re-establish adequate anticoagulation coupled with our extensive bedside experience with using concentrated clotting factors including the effectiveness of lower doses, we initiated this low-dose approach using PCC4 and developed an internal guideline to guide the process when requested. The purpose of this study is to assess our approach to using low-dose PCC4 for temporary warfarin reversal in LVAD patients undergoing invasive procedures.

## Methods

This is a retrospective, single-center, cohort review to describe the use of PCC4 for warfarin reversal in patients with LVADs undergoing elective and urgent/emergent procedures between September 2016 and September 2021 at the University of California, Davis Medical Center.

Patients 18 years of age or older were included for analysis if they had a continuous-flow LVAD and only received PCC4 for warfarin reversal prior to undergoing either an elective or urgent/emergent procedure. Patients were excluded if they were on any oral anticoagulant other than warfarin, received PCC4 administration for LVAD implant or explant, or received vitamin K with the PCC4. Collected data included patient demographics, LVAD type, concomitant antiplatelet therapy, procedure performed, PCC4 dose, preprocedure and postprocedure INR. Lactate dehydrogenase (LDH) values were collected based on the associated findings of Starling et al.^14^ Procedures and surgeries were classified into one of the 2 bleeding-risk categories: low to moderate and high using the CHEST 2022 Perioperative Management of Antithrombotic Therapy.^13^ Preprocedure lab values were collected within 8 hours of the procedure initiation and prior to PCC4 administration. Postprocedure lab values were collected within 24 hours following the completion of the procedure. Upon adequate hemostasis post procedurally, at the treating physicians’ discretion, use of an UF heparin bridge to a goal anti-Xa 0.3 to 0.5 unit/mL with warfarin may be utilized. Time to warfarin re-initiation and achievement of target INR were collected during the 30 day follow-up period. Target INR is defined as the patient’s outpatient goal INR, determined by the treating physician, and was collected for each patient through chart review. All patients were followed long term by our LVAD service.

The primary outcome was either pump thrombosis, venous thromboembolism, or ischemic stroke within 30 days of PCC4 administration. Chart review was performed to search for documentation of thrombotic events. The secondary outcome was bleeding defined by the Mechanical Circulatory Support Academic Research Consortium (MCS-ARC) type 3 to 5 within 30 days of the procedure.

Categorical and continuous variables were analyzed using descriptive statistics. Continuous variables are represented as median with interquartile range or median with overall range. The data were collected in compliance with the requirements of our Institutional Review Board/Human Subjects Research Committee.

### Administration Protocol

#### Low-bleeding risk procedures

**Figure 1.**
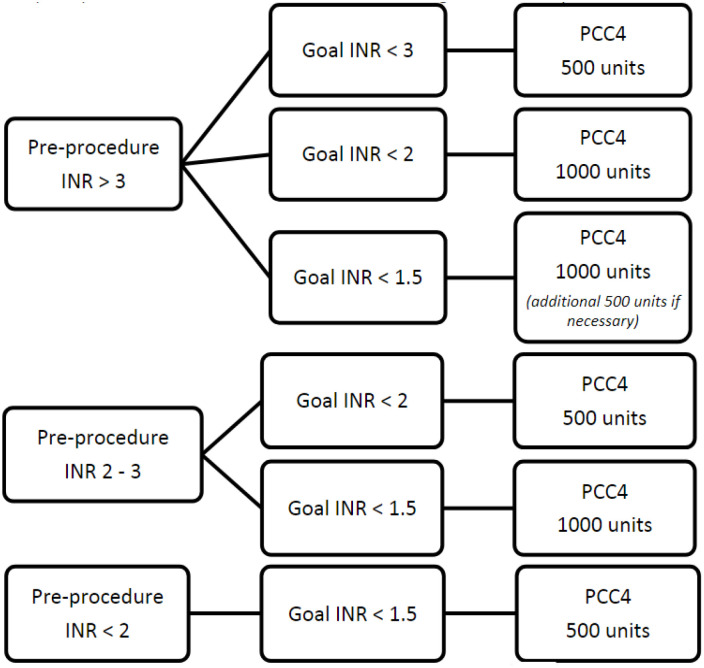
Low- to moderate-bleed risk procedure.

A preprocedure INR was obtained and categorized as either <2, 2-3, or >3. The physician performing the intervention determined the target INR acceptable for the procedure, which was then categorized as either an INR <3, <2, or <1.5. Preprocedure INR value along with desired INR goal was used to determine the PCC4 dose to be recommended. The PCC4 dose range was 500 to 1000 units with an optional additional 500 units available for patients with preprocedure INR >3 and procedural INR goal <1.5 (Figure 1).

#### High-bleeding risk procedures

**Figure 2.**
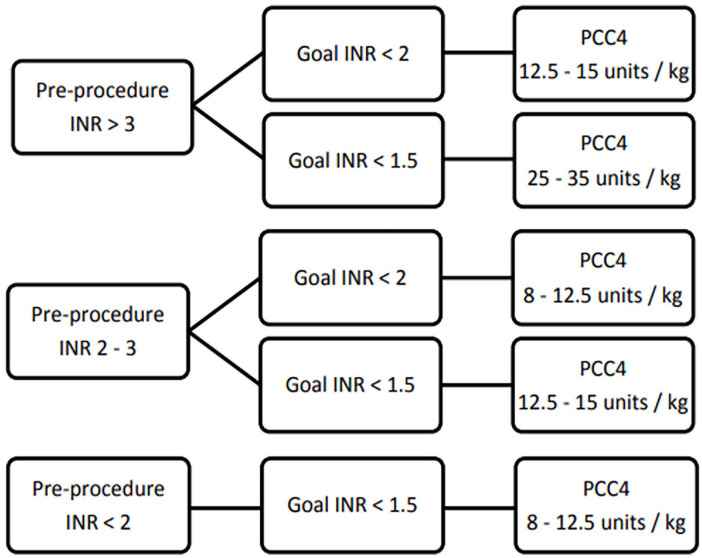
High-bleed risk procedure.

Similar to the low-bleeding risk protocol, we obtain preprocedure INR and categorize as either <2, 2-3, or >3. Preprocedure INR goal will be selected as either INR <2 or <1.5. The dose determination utilized a weight-based approach rounding to the nearest factor IX vial content with the option for an additional 500 units if INR not at or below procedural goal (Figure 2). The final PCC4 dose to be administered was at the discretion of the treating physician.

## Results

In total, 14 patients received 17 administrations of PCC4 for procedural warfarin reversal (Table 1). All patients were followed long term by our LVAD service. One patient received 3 administrations for 3 different procedures over the span of 9 months and 1 other patient received 2 administrations for 2 different procedures over the span of 2 years. Nine patients had HeartMate II, 3 HeartWare, and 2 HeartMate III devices. Esophagogastroduodenoscopy (EGD) and colonoscopy were the most common procedural indications for warfarin reversal. Emergent procedures requiring warfarin reversal compromised 23.5% (4/17) of the population.

**Table 1.** Cohort Characteristics.

Age, median (SD), years	72 (18)
Male, n (%)	11 (78.6)
Weight, mean (SD), kg	91.2 (27.8)
Device type, n	
HeartMate II	9
HeartWare	3
HeartMate III	2
PCC4 administrations, n	17
Emergent procedures	
Exploratory laparotomy^a^	1
Chest tube	2
Open reduction and internal fixation of femur^a^	1
Elective procedures	
Esophagogastroduodenoscopy	5
Colonoscopy	5
Transurethral resection of prostate^a^	1
Splenic artery embolization	1
Tooth extraction	1
Preprocedure INR, median (SD)	2.77 (1.01)
Postprocedure INR, median (SD)	1.66 (0.55)
Preprocedure LDH, median (SD)	203 (411)
Postprocedure LDH, median (SD)	207 (328)
Warfarin restart	
0-24 hours	5
24-48 hours	6
>48 hours	6
Target INR achieved	
0-<24 hours	2
24-48 hours	4
>48 hours	11
Aspirin, n	11

Median preprocedure INR and LDH were 2.77 and 203, respectively. The median PCC4 dose for all patients was 500 units or 6.6 units/kg (range = 4.2-14.1 units/kg) based on the Factor IX contents. Median PCC4 dose was 6 units/kg and 11.7 units/kg for low to moderate- and high-bleeding risk procedures, respectively. After the initial PCC4 administration during each procedural encounter, no additional doses of PCC4 were administered.

Abbreviations: INR, international normalized ratio; Kg, kilograms; LDH, lactate dehydrogenase; PCC, prothrombin complex concentrate; SD, standard deviation.

a High-bleeding risk procedures.

In the entire cohort of patients after roughly 39 minutes post-PCC4 dose, the median pre-PCC4 INR of 2.77 (standard deviation [SD] = 1.01) dropped to median 1.57 (SD = 0.15). Roughly, 8:53 hours postprocedure, the median INR was 1.66 (SD = 0.55). The median INR 22 hours postprocedure was 2.22 (SD = 1.49).

**Figure 3.**
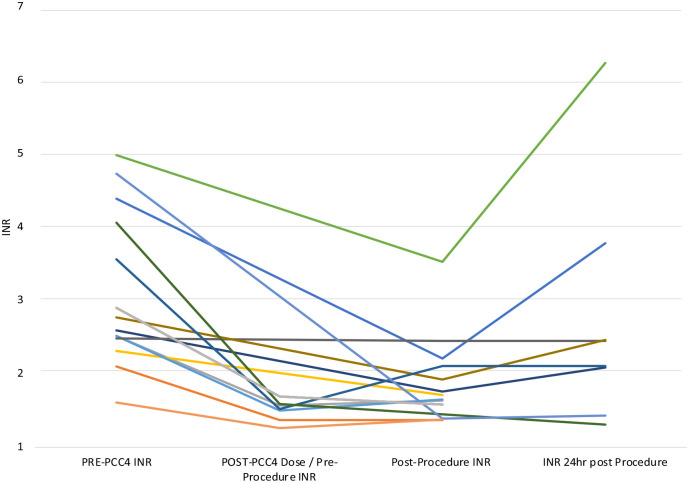
Low to moderate-bleeding risk procedures.

For the 14 low to moderate-bleeding risk procedures (Figure 3), PCC4 was administered at a median time of 1 hour prior to the procedure. The median preprocedural INR of 2.68 (SD = 1.05) dropped to an INR of 1.52 (SD = 0.14) 39 minutes post-PCC4 administration. The median postprocedure INR values were 1.71 (SD = 0.59) at 9:43 hours and 2.28 (SD = 1.61) at 23:04 hours.

**Figure 4.**
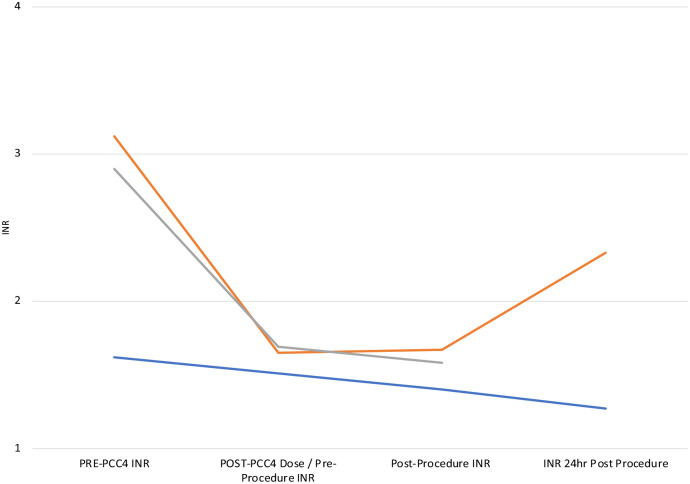
High-bleeding risk procedures.

For the 3 high-bleeding risk procedures (Figure 4), PCC4 was administered at a median time of 39 minutes prior to procedure. The median preprocedural INR of 2.90 (SD = 0.81) dropped to an INR of 1.67 (SD = 0.03) 10:49 hours post-PCC4 administration. The median INR values postprocedure were 1.58 (SD = 0.14) at 1:17 hours and 1.80 (SD = 0.75) at 18:35 hours.

Of the 17 PCC4 administration encounters, 11 were restarted on warfarin and 6 achieved target INR values within 48 hours of procedure end. There were no reported cases of pump thrombosis, venous thromboembolism, or stroke within our 30 day follow-up period. There was 1 case of MCS-ARC type 3a that occurred within the 30 day follow-up period.

## Discussion

We describe the use of PCC4 as a rapid means to transiently reverse the effects of warfarin in patients with continuous-flow LVADs undergoing elective or emergent procedures. Data and published experience evaluating the use of PCC4 in this manner are limited. Brown et al^15^ describe the use of low-dose PCC4 in 2 patients with HeartMate II devices undergoing procedures. Both patients were administered doses of PCC4 and vitamin K IV once. The first patient was given 16 units/kg (1648 units) PCC4 for an urgent percutaneous cholecystostomy. The preprocedure INR of 4.7 decreased to 2.3, 45-minutes post-PCC4 administration. The second patient presented with right occipital intraparenchymal hemorrhage and subdural hematoma. This patient was administered PCC4 11 units/kg (1058 units) resulting in the INR decreasing from 3.7 to 1.6. Both patients received heparin infusions postprocedure to bridge until therapeutic INR was achieved. Neither patient experienced signs of pump thrombosis or thrombotic event nor were there procedural or neurological complications.

Rimsans et al^16^ described that in 37 HeartMate II or HeartWare patients with 49 admissions who received an average dose of 22 units/kg, the mean INR was reversed from 2.9 to 1.7. Patients undergoing low-bleeding risk procedures received an average of 17 units/kg, whereas patients undergoing high-bleeding risk procedures were administered an average of 30 units/kg. Patients who received vitamin K accounted for 32% of the population. There were no observations of thrombotic events within 30 days and temporary reversal was deemed 96% effective. Our baseline INR values were overall lower than the 2 reports with similar values post-PCC4 administration. In our evaluation, we were able to include more patients not only with the HeartMate II device but also with HeartWare and HeartMate III devices. In addition, we were able to achieve procedural hemostasis utilizing lower doses of PCC4.

For low-bleeding risk procedures, we utilize a lower, fixed PCC4 dose regimen. We included patients undergoing emergent procedures and with HeartMate III devices. Our median weight-based dosing was 6.6 units/kg, which is lower than the doses previously described. We observed adequate procedural hemostasis utilizing this lower dosing regimen and no thrombotic events. The 1 case of bleeding was gastrointestinal in nature and occurred 19 days after the administration of PCC4 in a patient who experienced several gastrointestinal bleeds requiring transfusions in the past.

High-bleed risk procedures represent a minority of the cases we evaluated. However, additional PCC4 doses were not required for any of our patients, irrespective of the procedure bleed risk. Which suggests adequate procedural hemostasis was achieved with our weight-based high-bleed risk procedure PCC4 dosing regimen.

The use of PCC4 carries the fundamental concern of triggering thrombotic events. With our low, fixed dose PCC4 protocol, we were able to achieve preprocedural INR goals, hemostasis and reduce patient exposure to PCC4. Postprocedurally, once adequate hemostasis is achieved at the treating physicians’ discretion, we resume warfarin while bridging with heparin, goal anti-Xa 0.3-0.5 unit/mL, until target INR achieved. With our PCC4 dosing protocol, we were able to achieve target INR in a respectable amount of our patients within 48 hours postprocedure. We suspect this is in part due to less drastic INR reductions preprocedurally by administering low PCC4 doses.

In our high-bleeding risk procedures, we utilize a weight-based dosing strategy to a maximum dose of 35 units/kg. While patients who underwent high bleeding risk procedures represent a minority of the population evaluated, we were able to utilize the lower end of our weight-based strategy and achieve adequate procedural hemostasis and limit PCC4 exposure.

In the setting of low-bleeding risk procedures in patients on warfarin with LVADs, commonly the goal is to temporarily reverse patients to safely perform the procedure. Immediately following the procedure, the goal is to achieve target INR as soon as possible to prevent devastating thrombotic events, such as pump thrombosis. We were able to show that low-dose regimen of PCC4 with the option of heparin bridging until the target INR is re-established is effective at achieving procedural hemostasis and is not associated with thrombotic events.

There are several limitations of this evaluation to keep in mind; the majority of our patients were implanted with HeartMate II devices, single-center retrospective approach with a small sample size, and the lack of comparison group. We recognize that the patients received the HeartMate II device and recognize the technology of newer generation LVADs potentially having lower thrombotic rates. Given our lack of seeing any with the earlier LVAD versions would lead one to postulate using low-dose PCC4 as described might be a safe practice in newer generations LVAD devices. However, this is always the challenge when device technology moves faster than the pharmacotherapy.

Given the timeframe of which we evaluated patients, the majority of patients had already received their HeartMate II device and our institution was not routinely explanting and exchanging patients for HeartMate III devices. However, we would not expect to see an increase in thrombotic or bleeding events with our PCC4 dosing protocol had more HeartMate III patients been included based on the mechanical differences in pump designs.^17^ At the time of our evaluation, to our knowledge, this is the only analysis in which any HeartMate III patients were included for procedural warfarin reversal with PCC4. Relative to other cohort reviews that analyzed PCC4 dosing in LVAD patients, our sample size is comparable exemplifying the nuance of this patient population and clinical scenario.

## Conclusions and Relevance

This retrospective, single-center analysis suggests that a relative low dose of PCC4 for patients with LVADs undergoing procedures appears to be a safe and effective strategy for temporary warfarin reversal. The proposed procedural PCC4 dosing for LVAD patients appears to be 1 approach to guide clinicians to temporarily reverse warfarin. The value of minimizing preprocedural warfarin interruption and utilizing PCC4 for temporary reversal in relation to cost and length of stay should be evaluated in future studies. Additional multicenter, preferably randomized or cohorts are necessary to further describe the best approach to temporarily reversing warfarin.
